# Loculations and Associated Risk Factors of Childhood Pleural Tuberculosis

**DOI:** 10.3389/fped.2021.781042

**Published:** 2021-12-16

**Authors:** Jun-Li Wang, Ming Zhou, Yan-An Zhang, Mao-Shui Wang

**Affiliations:** ^1^Department of Lab Medicine, The Affiliated Hospital of Youjiang Medical University for Nationalities, Baise, China; ^2^Department of Lab Medicine, Longtan Hospital of Guangxi Zhuang Autonomous Region, Liuzhou, China; ^3^Department of Cardiovascular Surgery, Shandong Public Health Clinical Center, Cheeloo College of Medicine, Shandong University, Jinan, China; ^4^Shandong Key Laboratory of Infectious Respiratory Disease, Jinan, China; ^5^Department of Lab Medicine, Shandong Provincial Chest Hospital, Cheeloo College of Medicine, Shandong University, Jinan, China; ^6^Department of Lab Medicine, Shandong Public Health Clinical Center, Cheeloo College of Medicine, Shandong University, Jinan, China

**Keywords:** children, pleural tuberculosis, loculation, risk factor, empyema

## Abstract

**Background:** Pleural loculation in childhood pleural tuberculosis (TB) remains a problem in practice, it is usually associated with failure drainage. Therefore, to improve the management of childhood pleural TB, a retrospective study was conducted to identify the risk factors associated with loculated effusion in childhood pleural TB.

**Methods:** Between January 2006 and December 2019, consecutive children (≤15 years old) with tuberculous pleural effusion (definite and possible) were included for further analysis. The demographic, clinical, laboratory, and radiographic features were collected from the medical records. Univariate and multivariate logistic regressions were used to explore the factors associated with the presence of pleural loculation in children with pleural TB.

**Results:** A total of 154 children with pleural TB (definite, 123 cases; possible, 31 cases) were included in our study and then were classified as loculated effusion (*n* = 27) and non-loculated effusion (*n* = 127) groups by chest X-ray or ultrasonography. Multivariate analysis revealed that male gender (age-adjusted OR = 3.903, 95% CI: 1.201, 12.683), empyema (age-adjusted OR = 4.499, 95% CI: 1.597, 12.673), peripheral monocytes ≤0.46 × 10^9^/L (age-adjusted OR = 4.122, 95% CI: 1.518, 11.193) were associated with the presence of loculated effusion in children with pleural TB.

**Conclusion:** In conclusion, several characteristics, such as male gender, empyema, and peripheral monocyte count have been identified as risk factors for pleural loculation in children with pleural TB. Our findings may be helpful to improve the management of pleural loculation in childhood pleural TB.

## Introduction

Tuberculosis (TB) remains a serious threat to children health. Every year, an estimated 1 million children become sick with TB and most of these children are newly diagnosed or never treated (1). In addition, childhood TB is known as a paucibacillary form of TB disease and children might have extrapulmonary TB (2, 3), make the TB control in children more complicated. As a common extrapulmonary TB in adulthood, pleural TB was also present in children (4). In addition, unfortunately, during the past decade, the proportion of pleural TB among childhood TB showed an increasing trend (4). However, on the other side, the good news is that its overall treatment completion rate was up to 94.3% and no deaths (5).

Because few cases are confirmed by microbiological investigations (6), tuberculin skin test remains a useful tool in establishing the diagnosis of childhood pleural TB. Moreover, like adulthood pleural TB, childhood pleural TB could also develop into loculated effusion, even though effective anti-TB therapy is easily obtained (7). Loculations may impair pleural drainage and sequester the infectious agents. These areas of loculation can ultimately become a source of ongoing pleural sepsis (8). Although intrapleural fibrinolytic therapy is effective in the management of patients with loculated pleural effusion (9), a significant proportion of patients remain in no response to the therapy (9, 10). In addition, complications, such as chest pain may reduce the availability of fibrinolytic therapy (5). Besides these, due to the severity of the disease state, failure also occurred and needed a second procedure when patients were treated with fibrinolytics (11). Hence, surgical excision remains an important tool in the management of children with loculated pleural effusion (11, 12).

Until now, few studies have been conducted examining the risk factors associated with loculated effusion in childhood pleural TB. Therefore, in this study, we aimed to identify the risk factors regarding this may aid to initiate timely treatment for children at a high risk of pleural loculation.

## Materials and Methods

Between January 2006 and December 2019, consecutive children (≤15 years old) diagnosed as pleural TB were included in our study. Subsequently, the data, including demographic, clinical (symptoms, vital signs, comorbidities, etc.), laboratory (hematology, biochemistry, flow cytometry, etc.), and radiographic features (such as effusion site) were collected from the medical records, and these data were all collected on admission.

Definite pleural TB was defined as positive mycobacterial culture (sputum, pleural effusion, or pleural tissue), or suggested by pathological evidences (such as caseous necrosis, or Langhans' giant cells). Possible pleural TB was diagnosed based on the combinations of clinical symptoms and TB assays [TB RT-PCR, acid-fast bacilli (AFB) smear, or both]. Pleural effusion was defined as a collection of fluid limited by the diaphragm and the pleura. Then, the effusions were divided into loculated or free-flowing effusions by chest X-ray and ultrasonography (US). Loculated effusion was defined if an effusion met at least one following criteria: (1) failure to shift on decubitus film; (2) loculation demonstrated by chest X-ray or real-time chest US (13, 14). Usually, lateral and antero-posterior views of chest X-ray were performed in parallel and read by two experienced radiologists in our center. In addition, empyema was defined as grossly purulent pleural fluid (15). All patients were administered with first line anti-TB drugs (isoniazid, rifampicin, ethambutol, and pyrazinamide) for 9 months.

Statistical analysis was performed by SPSS version 16.0 (SPSS, Chicago, IL, USA). Continuous data were presented as mean ± standard deviation (SD) and categorical data were presented as number (percentage). Univariate logistic regression analysis was performed to assess risk factors responsible for the presence of loculated effusion and variables having a *P* < 0.1 were included for multivariate analysis to calculate the odds ratios (OR) and corresponding 95% confidence interval (CI) (16). To allow a better interpretation, continuous variables were transformed into categorical variables defined by cut-off value, which was determined by receiver operating characteristic curve (ROC) analysis. In addition, the accuracy of the multivariate model was evaluated by the Hosmer-Lemeshow goodness-of-fit test. All tests were 2-sided, and a *P* < 0.05 was considered statistically significant.

The study protocol was approved by the Ethics Committee of Shandong Provincial Chest Hospital (Jinan, China) and was conducted retrospectively at the hospital in accordance with the Declaration of Helsinki. Due to the retrospective nature and anonymous data collection, written informed consent was waived by the Ethics Committee of Shandong Provincial Chest Hospital.

## Results

### Patients Characteristics

The detailed patient characteristics were listed in Table 1 and Supplementary Table 1. During the study period, 1,577 children with TB were hospitalized at the hospital. Of them, a total of 154 (9.8%) children with pleural TB (definite, 123 cases; possible, 31 cases) were eligible for the study and then were classified as loculated effusion (*n* = 27, 17.5%) and non-loculated effusion (*n* = 127, 82.5%) groups.

**Table 1:** Univariate analysis of the demographic data associated with loculated effusion in childhood pleural TB.

		**Total (*n*)**	**Loculated effusion (*n*)**	**Non-loculated effusion (*n*)**	***P*-value**	**OR (95% CI)**
*N*		154	27	127		
Demographic characteristics						
	Age (years)	12.4 ± 3.3	12.0 ± 4.2	12.4 ± 3.1	0.575	
	Sex (male)	100 (64.9%)	23 (85.2%)	77 (60.6%)	0.021	3.734 (1.218, 11.442)
	Weight (Kg)	46.1 ± 16.2	44.7 ± 15.9	46.4 ± 16.3	0.608	
	Rural area	93 (60.4%)	18 (66.7%)	75 (59.1%)	0.464	
Signs and symptoms						
	Cough	84 (54.5%)	16 (59.3%)	68 (53.5%)	0.589	
	Fever	134 (87.0%)	23 (85.2%)	111 (87.4%)	0.756	
	Chest pain	69 (44.8%)	11 (40.7%)	58 (45.7%)	0.640	
	Dyspnea	42 (27.3%)	8 (29.6%)	34 (26.8%)	0.762	
	Sputum production	29 (18.8%)	4 (14.8%)	25 (19.7%)	0.558	
	Cavity	7 (4.5%)	3 (11.1%)	4 (3.1%)	0.091	3.844 (0.808, 18.283)
	Empyema	27	10 (37.0%)	17 (13.4%)	0.005	3.806 (1.497, 9.679)
Clinical Chemistry (serum)						
	Total protein (g/L)	68.7 ± 6.9	69.2 ± 6.8	68.6 ± 6.9	0.669	
	Albumin (g/L)	38.8 ± 4.8	38.9 ± 4.2	38.8 ± 4.9	0.936	
	Blood urea nitrogen (mmol/L)	3.9 ± 1.2	4.2 ± 1.1	3.8 ± 1.2	0.097	1.353 (0.947, 1.935)
	Creatinine (μmmol/L)	52.3 ± 15.3	55.9 ± 16.1	51.5 ± 15.0	0.185	
	Glucose (mmol/L)	4.8 ± 1.2	4.8 ± 0.7	4.9 ± 1.3	0.757	
	Lactate dehydrogenase (U/L)	219.8 ± 71.1	213.7 ± 79.8	220.9 ± 69.7	0.691	
Blood analysis						
	White blood cell (10^9^/L)	7.3 ± 2.6	6.8 ± 2.3	7.4 ± 2.6	0.248	
	Red blood cell (10^12^/L)	4.4 ± 0.5	4.5 ± 0.4	4.4 ± 0.5	0.307	
	Hemoglobin (g/L)	121.2 ± 14.2	123.3 ± 13.6	120.8 ± 14.3	0.397	
	Hematocrit	36.6 ± 3.9	37.0 ± 3.7	36.5 ± 4.0	0.507	
	Mean corpuscular volume (fL)	82.4 ± 5.1	81.7 ± 3.9	82.6 ± 5.3	0.430	
	Mean corpuscular hemoglobin (pg)	27.3 ± 2.1	27.2 ± 1.7	27.4 ± 2.2	0.773	
	Mean corpuscular hemoglobin concentration (g/L)	331.3 ± 12.7	332.8 ± 10.7	331.0 ± 13.1	0.506	
	Platelet (10^9^/L)	352.8 ± 129.0	350.8 ± 134.1	353.2 ± 128.4	0.929	
	Neutrophil (10^9^/L)	5.1 ± 6.2	4.1 ± 1.6	5.3 ± 6.8	0.121	
	Lymphocyte (10^9^/L)	1.7 ± 0.9	1.9 ± 1.4	1.7 ± 0.8	0.184	
	Monocyte (10^9^/L)	0.8 ± 0.4	0.6 ± 0.4	0.8 ± 0.4	0.054	0.254 (0.063, 1.021)
	Coefficient of variation of red cell distribution width (%)	13.9 ± 1.8	13.8 ± 1.3	13.9 ± 1.9	0.852	
	Erythrocyte sedimentation rate (mm/h)	40.8 ± 26.4	38.7 ± 28.9	41.2 ± 25.9	0.657	

*TB, tuberculosis; OR, odds ratio; CI, confidence interval*.

One hundred and eight (70.1%) patients were culture positive for TB, 57 (37.0%) were TB RT-PCR positive, 51 (33.1%) were found to have pathological evidence of TB, 15 (9.7%) were AFB positive, and 1 (0.6%) was Xpert positive. The mean age was 12.4 ± 3.3 years, mean weight was 46.1 ± 16.2 Kg, and 64.9% (100 patients) of them were male. Among the 154 children, 93 (60.4%) of them were from rural areas, 103 were tested for HIV status, and all were HIV-negative. The vital signs were recorded as follows: temperature, 37.2 ± 0.9°C; heart rate, 97.6 ± 16.0 beats/min (tachycardia, *n* = 52); respiratory rate, 22.5 ± 2.7 breaths/min; blood pressure, 111.4 ± 12.3/69.2 ± 8.5 mmHg.

Twenty (13.0%) patients were reported with a TB contact history and surgical treatment was given to 29 (18.8%) patients. Prior to our center, most of patients (91, 59.1%) were treated at a teaching hospital, and the mean frequencies of hospitalization were 2.0 ± 1.6. The mean time between first symptoms and anti-TB therapy was 12.4 ± 3.3 days. Fever (134, 87.0%) was the most frequent symptom, followed cough (84, 54.5%), chest pain (69, 44.8%), dyspnea (42, 27.3%), and sputum production (29, 18.8%). Among the 154 study patients, 89 (57.8%) of them had pulmonary TB, 13 (8.4%) had tuberculous lymphadenitis, 7 (4.5%) had miliary TB, 4 (2.6%) had tuberculous meningitis, and 3 (1.9%) had bronchial TB. Surgical treatment was given to 29 (18.8%) patients, including debridement (*n* = 4), decortication (*n* = 21), and thoracic surgery (*n* = 4). The successful treatment was seen in 94.8% (146/154) of children. There were eight relapse events (5.2%) and no death occurred during the study.

Other characteristics, such as radiographic findings, clinical chemistry analysis (serum or pleural effusion), blood cell analysis, and flow cytometry analysis, were summarized in Table 1 and Supplementary Table 1. There was no difference in these features (*P* > 0.05). However, due to *P* < 0.1, presence of lung cavity, blood urea nitrogen, and peripheral monocytes were included for further analysis.

### Univariate and Multivariate Analysis

Table 1 shows the results of univariate analysis by a comparison between loculated and non-loculated effusion groups. It was found that the presence of loculated effusion was associated with male gender (OR = 3.734, 95% CI: 1.218, 11.442) and empyema (OR = 3.806, 95% CI: 1.497, 9.679; all *P* < 0.05).

The corresponding optimal cut-off values were 0.42 mmol/L and 0.46 × 10^9^/L, for UREA and peripheral monocyte, respectively. Further multivariate analysis (Hosmer-Lemeshow goodness-of-fit test: *χ*^2^ = 4.832, df = 7, *P* = 0.680) revealed that male gender (age-adjusted OR = 3.903, 95% CI: 1.201, 12.683), empyema (age-adjusted OR = 4.499, 95% CI: 1.597, 12.673), peripheral monocytes ≤0.46 × 10 ^9^/L, age-adjusted OR = 4.122, 95% CI: 1.518, 11.193) were associated with the presence of loculated effusion in children with pleural TB (Table 2).

**Table 2:** Age-adjusted OR for risk factors associated with loculated effusion in childhood pleural TB.

	**Adjusted (age) OR**	***P*-value**
Male, female	3.903 (1.201, 12.683)	0.024
Empyema	4.499 (1.597, 12.673)	0.004
Monocytes (≤ 0.46 × 10^9^/L)	4.122 (1.518, 11.193)	0.005

*TB, tuberculosis; OR, odds ratio*.

## Discussion

Usually pleural loculations are associated with failed drainage. However, an early use of intrapleural fibrinolytics is advocated to prevent the development of loculations (17). Therefore, to identify the risk factors of loculated effusion in childhood pleural would aid to improve its management. In our study, several risk factors, such as male gender, empyema, and peripheral monocytes ≤0.46 × 10^9^/L were identified to have an association with the presence of loculated effusion in children with pleural TB. To our knowledge, this research is the first study investigating the risk factors associated with loculated effusion in children.

Until now, in terms of the treatment of loculated effusion, fibrinolytic therapy is recommended for the relief of distressing dyspnea due to loculations (18, 19). Surgical intervention could clear the loculations, drain serous fluid, and remove the fibrinous peel with satisfactory lung expansion. Although these have a number of limitations, such as invasive nature and increased adverse events, patients with loculated effusion would benefit from these approaches for early use (20, 21). Hence, our findings would be helpful to identify children at a high risk of loculated effusion, timely interventions are then given, and a significant improvement is performed in the management of childhood pleural TB.

In our study, male gender was identified as a risk factor of loculated effusion among children. The gender difference has been widely evaluated in research involving pleural diseases (such as PPE, pleural TB, and mesothelioma). For example, first, several observational studies suggested that more males were found with complicated PPEs and pleural empyema than female adults or children (22–24). Second, an epidemiological investigation of childhood TB demonstrated that pleural TB was found to be significantly more common in males (25). Third, besides the above mentioned, gender differences in outcomes were also validated in patients with mesothelioma and male individuals were found with a poor overall survival (26). Therefore, compared with female adults, males may have a high risk of the development of pleural TB and easily progressed into a serious presentation of pleural diseases, such as empyema and loculated effusion. However, until now, the biologically plausible mechanism of gender distribution among patients with pleural disease remains unknown and further research may be required.

Empyema is another risk factor for loculated effusion identified in childhood pleural TB. This has been well-characterized in other studies. For example, Shankar et al. reviewed the experience in the management of children empyema, and found that from 1986 to 1991, among 13 children with empyema, 10 of them had loculated collection (27). In a previous study, children with empyema were all documented having loculated pleural collections (28). Similar results were also reported in studies conducted by McLaughlin et al. (29), Gofrit et al. (30), and Bishwakarma et al. (31). However, in our study, among the 27 children with empyema, 10 of them (37.0%) had loculated effusion. Remarkably, the proportion in our study was lower than that reported in previous studies. Similarly, Soysal et al. found eight patients (7.7%) with loculated empyema among 104 children with pleural empyema (32). Therefore, the difference in the prevalence of loculated effusion in children with empyema may be associated with geographical distribution, improved TB treatment strategies, or eligibility criteria for subjects included.

Interestingly, in the study, low peripheral monocyte count was also found to be associated with the presence of loculated effusion. This may be caused by inflammation responses involving pleural loculation and peripheral blood monocytes may be then recruited and migrated into the pleural space. Several explanations may be responsible for it: (1) Mesothelial cells have the ability to release various mediators and proteins, such as monocyte chemotactic peptide (MCP) (33). Hence, peripheral monocytes may be then recruited by MCP into pleural space (34). (2) Due to excessive production of inflammatory cytokines, monocytes engaged in the pathogenesis of pleural TB, peripheral monocytes are then stimulated and recruited (35). Moreover, loculation may strengthen the inflammatory response and take part in the stimulation of monocytes. (3) The interplay between MCP and cytokines may make an increased production of MCP, and then peripheral monocyte is stimulated and recruited (36, 37).

The study has several limitations and necessary caution should be taken. First, retrospective design is an evident limitation of this study, prospective multicenter studies are warranted to confirm these findings. Second, due to the observational and exploratory nature, the results may not be generalized to other settings. Third, the sample size is relatively small. Therefore, sample selection bias should be paid attention. Fourth, our study has a long research period, due to improvement in medical care, a possible bias which may influence our results may be made. Meanwhile, for this reason, a prospective study is hard to perform and then a retrospective method may be appropriate for this study.

## Conclusions

Our findings suggest that pleural loculation in childhood pleural TB remains a problem in practice. Further analysis showed that several characteristics, such as male gender, empyema, and low peripheral monocyte count have been identified as risk factors for the presence of loculation in children with pleural TB. These findings may aid to initiate appropriate treatment earlier and help to improve the management of pleural loculation.

## Data Availability Statement

The original contributions presented in the study are included in the article/Supplementary Material, further inquiries can be directed to the corresponding authors.

## Ethics Statement

This study was conducted at the Shandong Provincial Chest Hospital and confronted for the Helsinki Declaration. The study protocol was approved by the Ethics Committee of Shandong Provincial Chest Hospital. Due to the retrospective nature of this investigation and the anonymous nature of the data collection, this retrospective study was exempt from the need for written informed consent by the Ethics Committee of Shandong Provincial Chest Hospital.

## Author Contributions

J-LW, Y-AZ, and M-SW designed the study. M-SW and MZ collected and analyzed the data. Y-AZ and M-SW wrote the initial manuscript. All authors read and approved the final manuscript.

## Funding

This work was supported by the Science Research and Technology Development Plan of Baise City (20203405) and Liuzhou City (2020NBAD0803).

## Conflict of Interest

The authors declare that the research was conducted in the absence of any commercial or financial relationships that could be construed as a potential conflict of interest.

## Publisher's Note

All claims expressed in this article are solely those of the authors and do not necessarily represent those of their affiliated organizations, or those of the publisher, the editors and the reviewers. Any product that may be evaluated in this article, or claim that may be made by its manufacturer, is not guaranteed or endorsed by the publisher.
