# Supplementary material for: Loculations and Associated Risk Factors of Childhood Pleural Tuberculosis
Source: Front Pediatr. 2021 Dec 16;9:781042. doi: 10.3389/fped.2021.781042 (PMC8716631; doi:10.3389/fped.2021.781042)
Supplement: Supplementary file 1 [file Table_1.docx]

| Supplementary Table 1. Univariate analysis of the demographic data associated with loculated effusion in childhood pleural TB. | | | | | |
| --- | --- | --- | --- | --- | --- |
|  | | Total (n) | Loculated effusion (n) | non-Loculated effusion (n) | P value |
| N | | 154 | 27 | 127 |  |
| Vital Signs | |  |  |  |  |
|  | Temperature (℃) | 37.2±0.9 | 37.1±0.8 | 37.3±0.9 | 0.425 |
|  | Heart rate (beats/min) | 97.6±16.0 | 97.7±17.6 | 97.6±15.7 | 0.984 |
|  | Respiratory rate (breaths/min) | 22.5±2.7 | 22.7±2.3 | 22.5±2.8 | 0.676 |
|  | Systolic pressure (mmHg) | 111.4±12.3 | 112.9±11.4 | 111.1±12.4 | 0.514 |
|  | Diastolic pressure (mmHg) | 69.2±8.5 | 69.5±6.9 | 69.1±8.9 | 0.851 |
| Medical history | |  |  |  |  |
|  | Contact history of TB | 20 (13.0%) | 3 (11.1%) | 17 (13.4%) | 0.750 |
|  | Transferred times | 2.1±1.0 | 2.3±1.0 | 2.0±1.0 | 0.232 |
|  | Transferred from a teaching hospital | 91 (59.1%) | 16 (59.3%) | 75 (59.1%) | 0.984 |
|  | Frequencies of hospitalization | 2.0±1.6 | 1.9±1.7 | 2.0±1.6 | 0.694 |
|  | Treatment delay (days) | 61.8±134.6 | 43.2±44.9 | 65.7±146.6 | 0.427 |
|  | Surgical treatment | 29 (18.8%) | 8 (29.6%) | 21 (16.5%) | 0.120 |
| Effusion sites | |  |  |  |  |
|  | Left | 62 (40.3%) | 13 (48.1%) | 49 (38.6%) | 0.359 |
|  | Right | 74 (48.1%) | 12 (44.4%) | 62 (48.8%) | 0.680 |
|  | Both | 18 (11.7%) | 2 (7.4%) | 16 (12.6%) | 0.451 |
| Comorbidity | |  |  |  |  |
|  | Pulmonary TB | 89 (57.8%) | 14 (51.9%) | 75 (59.1%) | 0.492 |
|  | Bronchial tuberculosis | 3 (1.9%) | 0 (0%) | 3 (2.4%) | 0.999 |
|  | Tuberculous lymphadenitis | 13 (8.4%) | 3 (11.1%) | 10 (7.9%) | 0.585 |
|  | Tuberculous meningitis | 4 (2.6%) | 0 (0%) | 4 (3.1%) | 0.999 |
|  | Milliary TB | 7 (4.5%) | 1 (3.7%) | 6 (4.7%) | 0.818 |
| Clinical Chemistry (pleural effusion) | |  |  |  |  |
|  | Total Protein (g/L) | 48.5±7.2 | 49.0±5.5 | 48.4±7.5 | 0.807 |
|  | Total Bilirubin (mmol/L) | 8.6±5.6 | 8.7±3.9 | 8.6±5.9 | 0.920 |
|  | Adenosine deaminase (U/L) | 60.3±28.8 | 61.8±25.1 | 60.0±29.6 | 0.837 |
|  | Glucose (mmol/L) | 3.3±1.5 | 2.9±1.4 | 3.4±1.6 | 0.316 |
|  | Lactate dehydrogenase (U/L) | 876.7±642.8 | 785.3±418.2 | 895.0±679.7 | 0.561 |
|  | Amylase (U/L) | 29.6±10.4 | 30.2±9.9 | 29.5±10.6 | 0.803 |
| Flow cytometry | |  |  |  |  |
|  | CD19+ (%) | 24.8±20.1 | 22.2±13.0 | 25.6±21.7 | 0.565 |
|  | CD3+ (%) | 62.4±13.7 | 61.0±13.4 | 62.7±13.8 | 0.664 |
|  | CD3+CD4+ (%) | 33.1±9.1 | 33.9±9.8 | 32.9±9.0 | 0.718 |
|  | CD3+CD8+ (%) | 24.0±12.0 | 23.7±11.4 | 24.1±12.3 | 0.902 |
|  | CD3-CD16+CD56+ (%) | 11.8±6.2 | 13.1±7.9 | 11.4±5.7 | 0.359 |
|  | CD4+/CD8+ | 2.8±3.9 | 2.8±4.3 | 2.8±3.9 | 0.988 |
| TB, tuberculosis; OR, odds ratio; CI, confidence interval. | | | | | |
